# Generation of B7‐H3 isoform regulated by ANXA2/NSUN2/YBX1 axis in human glioma

**DOI:** 10.1111/jcmm.18575

**Published:** 2024-07-24

**Authors:** Yifen Shen, Chunfang Ma, Xiangxiang Li, Xiaosong Li, Yuxiang Wu, Tao Yang, Yanping Hu, Chao Liu, Hao Shen, Pin Guo, Yihang Shen

**Affiliations:** ^1^ Central Laboratory, Suzhou Bay Clinical College Xuzhou Medical University, Suzhou Ninth People's Hospital Suzhou Jiangsu China; ^2^ Clinical Laboratory Suzhou Ninth People's Hospital Suzhou Jiangsu China; ^3^ Department of Anorectal Surgery Suzhou Ninth People's Hospital Suzhou Jiangsu China; ^4^ Department of Pathology Suzhou Ninth People's Hospital Suzhou Jiangsu China; ^5^ Department of Medical Cosmetology, Suzhou Ninth People's Hospital Soochow University Suzhou Jiangsu China; ^6^ Department of Molecular Pathology The Affiliated Cancer Hospital of Zhengzhou University, Henan Cancer Hospital Zhengzhou Henan China; ^7^ Department of Neurosurgery The Affiliated Hospital of Qingdao University Qingdao Shandong China

**Keywords:** 2Ig isoform, ANXA2, B7‐H3, NSUN2, RNA methylation, YBX1

## Abstract

In recent years, in the development of emerging immunotherapy, B7‐H3 is also termed as CD276 and has become a novel chimeric antigen receptor (CAR)‐T target against glioma and other tumours, and aroused extensive attention. However, B7‐H3 has three isoforms (2, 3 and 4Ig) with the controversial expression and elusive function in tumour especially glioma. The current study mainly focuses on the regulatory factors and related mechanisms of generation of different B7‐H3 isoforms. First, we have determined that 2Ig is dominant in glioma with high malignancy, and 4Ig is widely expressed, whereas 3Ig shows negative expression in all glioma. Next, we have further found that RNA binding protein annexin A2 (ANXA2) is essential for B7‐H3 isoform maintenance, but fail to determine the choice of 4Ig or 2Ig. RNA methyltransferase NOP2/Sun RNA methyltransferase 2 (NSUN2) and 5‐methylcytosine reader Y‐box binding protein 1 (YBX1) facilitate the production of 2Ig. Our findings have uncovered a series of factors (ANXA2/NSUN2/YBX1) that can determine the alternative generation of different isoforms of B7‐H3 in glioma. Our result aims to help peers gain a clearer understanding of the expression and regulatory mechanisms of B7H3 in tumour patients, and to provide better strategies for designing B7H3 as a target in immunotherapy.

## 1 INTRODUCTION

Glioma, one of the most aggressive malignant tumours in humans, accounts for approximately 40% primary intracranial tumours.^1^ Gliomas can be categorized as low‐grade or high‐grade tumours, in which high‐grade gliomas make up 90%.^2^ Despite advances in diagnostic and treatment approaches in recent years including surgical resection, chemotherapy, radiotherapy, the survival rate of glioma patients still remains unsatisfactory, especially for glioblastoma (GBM). The overall survival time after GBM diagnosis is only 14–15 months. The poor prognosis of glioma patients is mainly attributed to the diffuse infiltration of individual or clustered tumour cells into brain tissue. Immunotherapy as a new treatment methods currently holds a leading position in cancer care. Chimeric antigen receptors (CARs) are genetically synthetic immunoglobulin T cell receptor molecules that can recognize specific antigens and activate T cells.^3^ A novel CAR‐T cell product targeting CD276 (also called B7‐H3), a member of the B7/CD28 family, has been determined the feasibility of antitumor activity against glioma ex vivo and in vivo studies^4^, ^5^ as well as several cases of preliminary clinical trial.^6^

B7‐H3 is one member of peripheral membrane protein B7 families that participate in co‐stimulation of immune cells through an immune checkpoint signal.^7^ B7‐H3 can promote the activation of T cells and IFN‐γ production. Nevertheless, the immunosuppressive effects and cellular cytotoxicity of B7‐H3 on CD4^+^ and CD8^+^ T cells are also reported.^8^, ^9^ Therefore, the regulatory role of B7‐H3 on immune cells has been a subject of ongoing debate.

On the other hand, B7‐H3 is broadly expressed in various malignant tumours, and correlates with poorer prognosis in cancer patients.^10^, ^11^ To date, three isoforms of human B7‐H3 has been identified. The longest isoform (4Ig B7‐H3) contains two identical pairs of IgV‐like and IgC‐like domains, the shortest isoform (2Ig B7‐H3) includes only one pair, whereas the intermediate isoform (3Ig B7‐H3) lacks the first IgV‐like domain. Glioma, for instance, is respectively reported to highly expresses 4Ig^12^ and 2Ig B7‐H3^13^ by two independent researches. Additionally, 4Ig B7‐H3 is mainly detected in newly‐diagnosed GBMs, while 2IgB7‐H3 is specifically expressed in non‐cancerous brain tissue and highly expressed in recurrent GBMs.^14^ More extensively, 4Ig B7‐H3 is the predominant in human cancers,^15^ while the majority of 2Ig B7‐H3 is found in acute myeloid leukaemia.^16^ 4Ig and 2Ig B7‐H3 appear to have a similar inhibitory function,^17^ and 3Ig B7‐H3 is never investigated. Most studies on B7‐H3 have ignored the different functions and biological significance of these isoforms, instead lumping them together. In a word, the different roles of B7‐H3 isoforms in responding to immune system are largely undetermined.

In current research, we focus on different B7‐H3 isoforms in glioma, and investigate the regulatory mechanism of isoform splicing. Our findings will help people to further understand the transcription regulation of B7‐H3 in glioma, and improve the receptor structure to produce more effective CAR‐T cells for cancer treatment.

## 2 MATERIALS AND METHODS

### 2.1 Clinical specimen collection

Twenty‐four primary glioma specimens were obtained by the department of neurosurgery of The Affiliated Hospital of Qingdao University from January 2022 to January 2023. Patients have increased intracranial pressure, neurological and cognitive dysfunction and seizures. CT and MRI showed that the characteristic density including calcification, haemorrhage and cystic changes, as well as the lesion involved site, edema status and occupying effect. The information of pathological classification and WHO grade are listed in Table 1.

**TABLE 1:** The basic information of 24 primary glioma specimens were listed.

Case number	Pathological classification	WHO grade	Gender	Age
1	Pilocytic astrocytoma	I	M	13
2	Embryonal dysplastic neuroepithelial tumour	I	M	47
3	Papillary glial neuron tumour	I	M	24
4	Diffuse astrocytoma (IDHc mutation)	II	F	46
5	Diffuse astrocytoma (IDH mutation)	II	M	64
6	Diffuse astrocytoma (IDH mutation)	II	M	57
7	Oligodendroglioma (IDH mutation)	II	F	46
8	Oligodendroglioma (IDH mutation)	II	M	33
9	Anaplastic astrocytoma	III	M	69
10	Anaplastic astrocytoma	III	F	64
11	Anaplastic oligodendroglioma	III	F	38
12	Anaplastic oligodendroglioma	III	M	51
13	Glioblastoma (IDH wild type)	IV	F	67
14	Glioblastoma (IDH wild type)	IV	M	73
15	Glioblastoma (IDH wild type)	IV	M	57
16	Glioblastoma (IDH wild type)	IV	M	47
17	Glioblastoma (IDH wild type)	IV	F	48
18	Glioblastoma (IDH wild type)	IV	F	55
19	Glioblastoma (IDH wild type)	IV	M	52
20	Glioblastoma (IDH mutation)	IV	M	59
21	Glioblastoma (IDH mutation)	IV	F	44
22	Glioblastoma (IDH mutation)	IV	M	51
23	Glioblastoma (IDH mutation)	IV	F	64
24	Gliosarcoma	IV	M	29

### 2.2 Cell culture

A172, LN229, U87MG, U251MG and U373MG (National Collection of Authenticated Cell Cultures) were cultured in DMEM (HyClone, USA) containing with 10% FBS (HyClone) and 1 × penicillin–streptomycin antibiotics (MedChemExpress, China). The nucleotides of siRNA against Anxa2 (5′‐GACCAACCGCAGCAAUGCATT‐3′, 5′‐UGCAUUGCUGCGGUUGGUCTT‐3′),^18^ Nsun2 (5′‐CAGUGGAAGGUAAUGACGAAATT‐3′, 5′‐UUUCGUCAUUACCUUCCACUGTT‐3′)^19^ and Ybx1 (5′‐GGUUCCCACCUUACUACAU‐3′, 5′‐AGAAGGUCAUCGCAACGAA‐3′)^20^ referred as previously described were synthesized by Tsingke Biotech (China), and transfected into cells with 60% density using Hieff Trans siRNA/miRNA Kit (YEASEN, China). PCDNA3.1 vector of Anxa2, wild type and mutant Nsun2, and Ybx1 fusing with Flag tag were prepared by Tsingke Biotech, and transfected into cells with 60% density using Hieff Trans Kit (YEASEN).

### 2.3 RT‐PCR

Total RNA of cells was isolated using MolPure Cell RNA Kit (YEASEN). 800 ng RNA was reversed transcribed into cDNA using Hifair III Reverse Transcriptase Kit (YEASEN). Primer sequence of P1: 5′‐GCAGGGGCAGCCTTCCACCACGGG‐3′, 5′‐TCAGGCTATTTCTTGTCCATCATCT‐3′; P2: 5′‐TGGGCCGCGTCCCTGAGTC‐3′, 5′‐CATAGGCTGCCCTGTGATGGTGAC‐3′ for three different isoforms of B7‐H3. The annealing temperature of P1 and P2 were 52°C and 58°C. PCR was carried on using 2× HieffCanace PCR Master Mix (YEASEN). PCR products were separated through 1% agarose gel by electrophoresis at 120 V for 1 h. Images were captured using Tanon Cgenu Dog 5200T System (Tanon, China).

### 2.4 Western blot

Glioma tissues or cells at least 5 × 10^6^ cell count were lysated using 1 mL ice‐cold RIPA Lysis Buffer (YEASEN) for 30 min with occasional vortex. After 12,000× *g* centrifugation for 30 min, the protein supernatant was transferred into a new Eppendorf tube, and quantified by BCA Protein Quantification Kit (YEASEN). 40 μg total protein was loaded on 8% SDS‐PAGE gel. Primary antibodies against B7‐H3 (1: 2000, Cat. no. 58798, CST, USA), ANXA2 (1: 2000, Cat. no. 8235, CST), NSUN2 (1: 2500, Cat. no. 44056, CST), YBX1 (1: 2000, Cat. no. 9744, CST), β‐actin (1: 5000, Cat. no. 4967, CST) and Flag (1: 1000, Cat. no. 14793, CST) were used. Other conditions such as washing and incubation are consistent with regular western blot.^21^, ^22^

### 2.5 RNA pull down by probe and mass spectrum

Biotinylated DNA probe against the domain spanning 4th exon and 4th intron of pre‐mRNA of B7‐H3 was synthesized by Tsingke Biotech. 5 × 10^6^ cells were lysated using 1 mL ice‐cold RIPA buffer (0.5% NP40 as the detergent) for 30 min with occasional vortex. After 12,000× *g* centrifugation for 30 min, the protein supernatant was transferred into a new Eppendorf tube. 200 μmol probes were pre‐incubated with 20 μL Streptavidin Magbeads (YEASEN) at 4°C for 30 min (the amount for one experiment). Then cell lysate and probe/beads were mixed at 4°C for 2 h with slow rotation. After washing accordingly, 100 μL Elution buffer was added to harvested protein at boiled water bath for 10 min. Supernatant was transferred into another new Eppendorf tube for mass spectrum after 12,000× *g* centrifugation. HPLC‐MS/MS method provided by OE Biotech (China) was used to identify the binding proteins on B7‐H3 and semi‐quantify the difference among different groups.

### 2.6 RIP‐qPCR

5 × 10^6^ cells were harvested, resuspended in nuclear isolation buffer (Thermo Fisher Scientific, USA) on ice for 30 min with occasional vortex. The pellet nuclei were centrifuged with 14,000 rpm for 15 min and resuspended by wash buffer. 90% nuclei were incubated with 1 μg antibody of ANXA2 (Cat. no. H00000302‐M02, Novus Biologicals, USA) overnight and 40 μL rProtein A/G MagBeads (YEASEN) 2 h by slow rotation at 4°C while the rest of 10% were harvested as input. The pellet beads were centrifuged by 3000 rpm 3 min, washed three times. The RNA in the input and pellet beads were isolated and reverse transcribed into cDNA. See “RT‐PCR” for relevant reagents and experimental procedures. Quantitative PCR was performed using Hieff UNICON advanced qPCR SYBR Master Mix (YEASEN). Primers sequence compassed the region around translate start site of B7‐H3: 5′‐ATTCGGGCCGGGCCTCGCTGCGGCG‐3′, 5′‐GAGGCAGAACCACAGTGCTCCCAGG‐3′.

### 2.7 meRIP‐qPCR

See “RT‐PCR” for relevant reagents and experimental procedures of total RNA isolation. 1 μg 5mC antibody (Cat. no. NBP2‐42814, Novus Biologicals) was incubated with 2 μg RNA overnight. See RIP‐qPCR for other steps for IP, RNA isolation and qPCR.

### 2.8 IP‐WB

1 × 10^7^ cells were disintegrated using 1 mL ice‐cold RIPA Lysis Buffer on ice for 30 min, then centrifuged to harvest the supernatant, and mixed with 1 μg antibodies against Flag or rabbit IgG for slowly rotating overnight at 4°C, and 40 μL rProtein A/G MagBeads for additional 2 h incubation. See “RIP” and “WB” for other steps such as washing, elution and WB.

### 2.9 RNA‐EMSA

Methylated or unmethylated RNA molecule (397 nt) compassed the 4th exon and 4th intron of B7‐H3 was synthesized by Tsingke Biotech. Recombinant human ANXA2 (Cat. no. ab93005, Abcam, USA) and YBX1 (Cat. no. ab187443, Abcam). RNA‐EMSA was performed using the LightShift Chemiluminescent RNA EMSA Kit (Thermo Fisher Scientific).

### 2.10 Statistical analysis

All experimental data were processed and analysed using SPSS 22.0 statistical software (IBM Corp. USA). The measurement data were expressed as mean ± standard deviation. One‐way ANOVA was used for comparison between multiple groups. The *p*‐value less than 0.05 was considered as statistical significance.

## 3 RESULTS

### 3.1 Characterization of B7‐H3 expression in glioma

**FIGURE 1:**
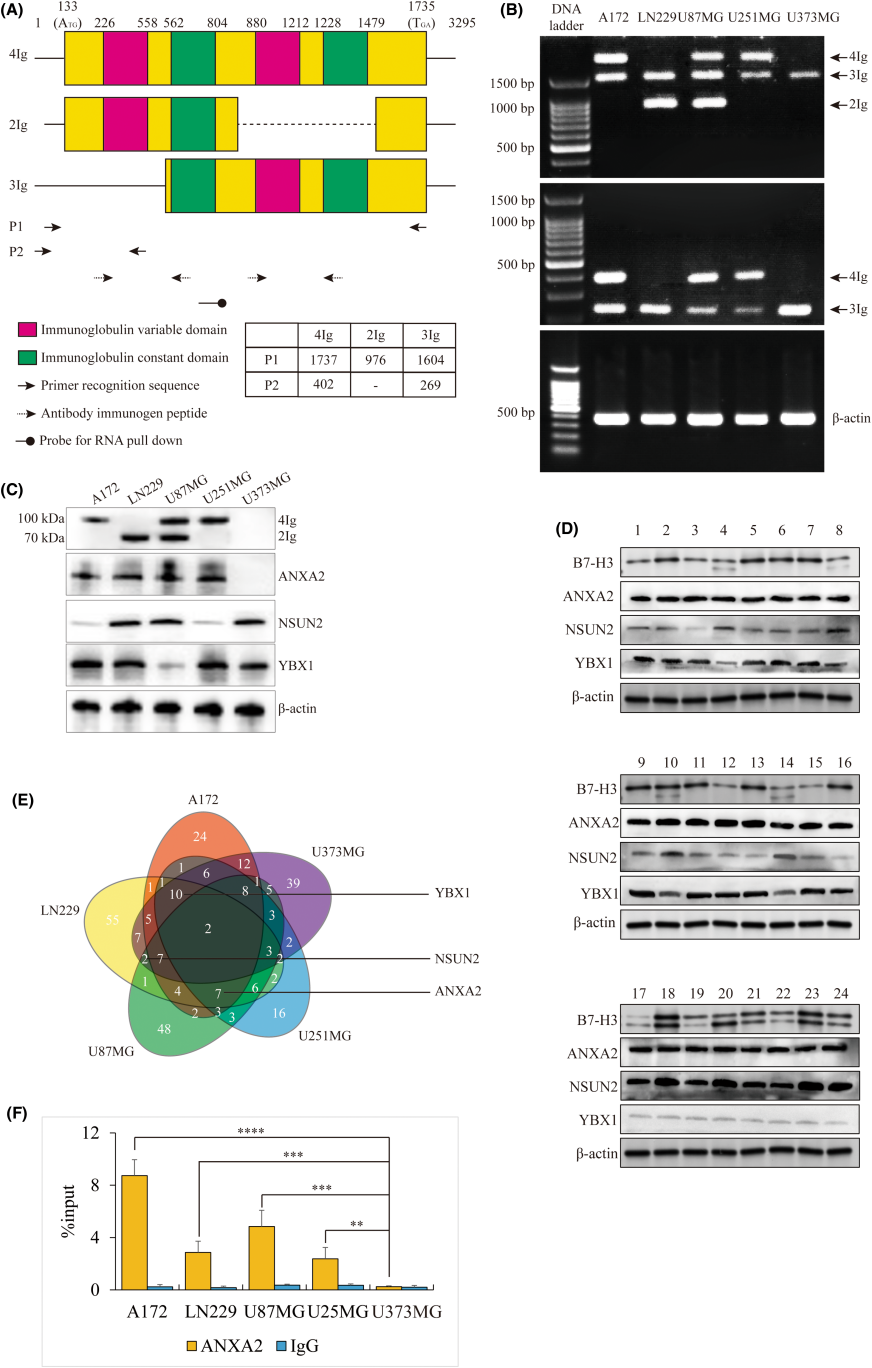
Characterization of different isoforms of B7‐H3 expression. (A) Cartoon displays the transcript length and basic structure information of the different isomers of B7H3, as well as primers, antibodies and probes used in this study. The table at the bottom right gives the length of the different isoform PCR products, which can be used as a reference for all subsequent RT‐PCR results. (B) RT‐PCR show the PCR products of B7‐H3 isoform in five glioma cell lines used by P1 primers (top), P2 primers (middle) and β‐actin primers (bottom). (C) WB assay shows the protein bands of B7‐H3 isoform, ANXA2, NSUN2 and YBX1 in five glioma cell lines. The band locations of 4Ig and 2Ig and the absence of 3Ig are inferred by RT‐PCR. (D) WB assay shows the protein bands of B7‐H3, ANXA2, NSUN2 and YBX1 in 24 glioma tissues. The double bands represent 4Ig and 2Ig according to the protein size in (C). (E) Venn diagram shows the binding proteins on B7‐H3 pre‐mRNA via probe pull down and mass spectrum. YBX1, NSUN2 and ANXA2 are highlighted in different intersections. (F) Enrichment of ANXA2 on B7‐H3 in five glioma cells by RIP‐qPCR. The statistical significances compared between other four cells and U373MG via one‐way ANOVA are indicated by asterisk. ****p < 0.0001, ***p < 0.001 and **p < 0.01.

Initially, five glioma A172, LN229, U87MG, U251MG and U373MG cell lines were picked up to investigate the expression of B7‐H3 by RT‐PCR and WB assay. Three isoforms of B7‐H3 and the corresponding primers and antibody recognition regions were represented in Figure 1A. Except U373MG, other four cell lines all showed the transcripts of two of three variants (Figure 1B). WB assay showed that 2Ig B7‐H3 was shown in LN229 and U87MG, while 4Ig B7‐H3 was expressed in A172, U87MG and U251MG cells. Inconsistent with the results of RT‐PCR, 3Ig B7‐H3 was failed to translate into protein in any cells (Figure 1C), indicating a complicated posttranscriptional regulation of B7‐H3 in different glioma cells (LN229 and U87MG). Furthermore, the protein expression of B7‐H3 was extended to profile in 24 glioma specimens in vivo (Table 1). It was notable that the expression of 4Ig B7‐H3 was dominant in all glioma, whereas, 2Ig B7‐H3 was mainly abundant in those glioma with high malignancy (no. 17–24, WHO Grade I and II). 3Ig B7‐H3 was barely expressed in glioma tissue (Figure 1D). Only the expression of 2Ig B7‐H3 showed a weakly positive correlation with tumour malignancy (*r* = 0.319, *p* = 0.043). Our first finding characterized the expression of B7‐H3 in glioma ex vivo and in vivo, and determined the presence of the inconsistent transcription and translation of different type of B7‐H3 in glioma.

### 3.2 ANXA2 is required for generation of B7‐H3 isoforms in glioma cells

In order to investigate the production machinery of different isoforms of B7‐H3, we harvested the proteomics binding on pre‐mRNA of B7‐H3 using DNA probe in these five glioma cell lines (Figure 1A). By mass spectrum, we noticed one RNA binding protein (RBP), annexin A2 (ANXA2) was found to interact with B7‐H3 transcripts in all glioma cells except U373MG (Figure 1E). Notably, ANXA2 was shown not to be expressed in U373MG cells (Figure 1C), whereas ANXA2 was robustly expressed in all glioma specimens in vivo (Figure 1D). Moreover, the robust interaction between ANXA2 and B7‐H3 transcripts in four glioma cells compared to U373MG was verified by RIP‐qPCR (Figure 1F). Given the absence of B7‐H3 isoform in U373MG, ANXA2 was speculated to play an important role in determining the translation of B7‐H3.

**FIGURE 2:**
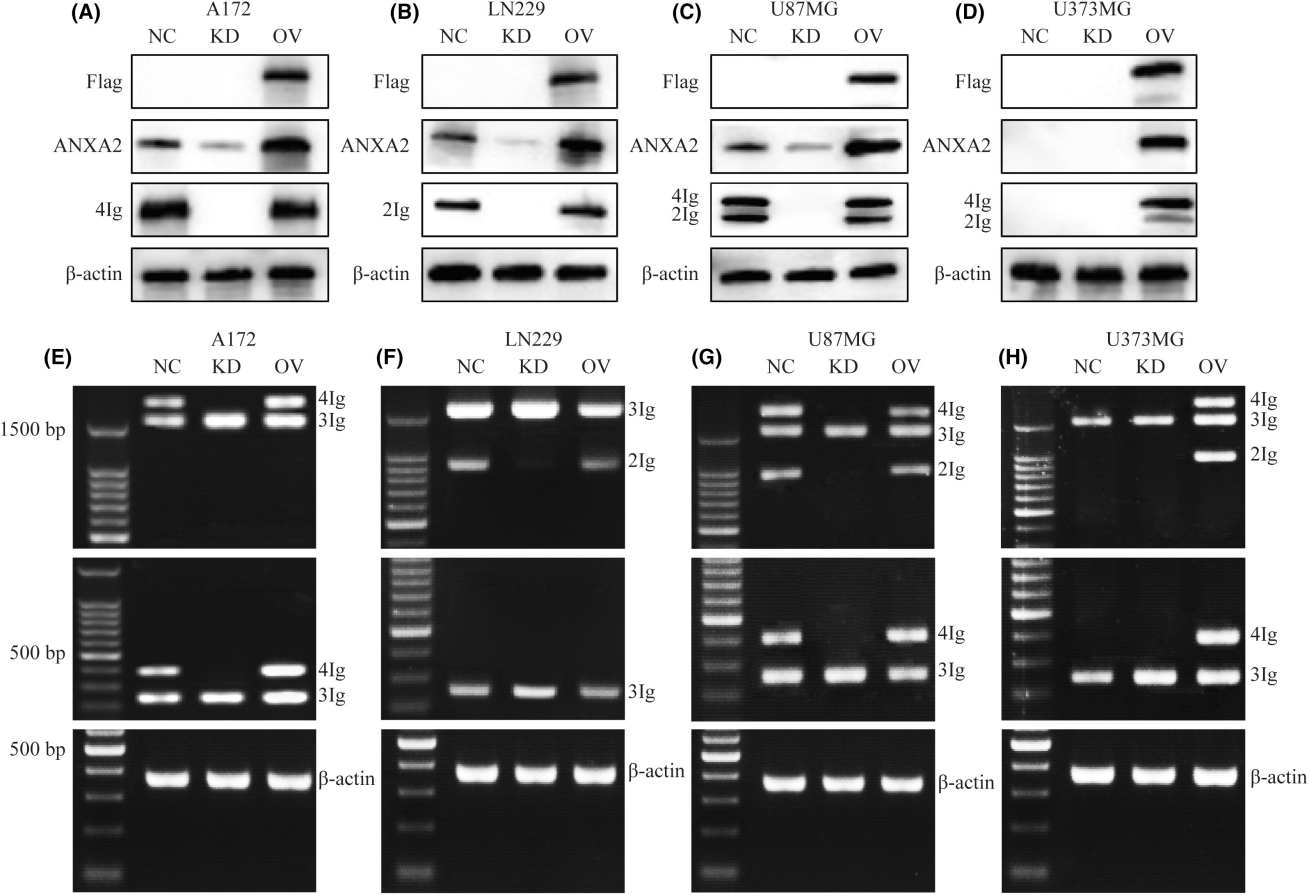
The effect of ANXA2 on the expression of different B7‐H3 isoforms. (A–D) WB assay shows the expression of 4Ig and 2Ig affected by knockdown or over‐expression of ANXA2 in A172 (A), LN229 (B), U87MG (C) and U373MG cells (D). (E–H) RT‐PCR show the PCR products of B7‐H3 isoform affected by knockdown or over‐expression of ANXA2 in A172 (E), LN229 (F), U87MG (G) and U373MG cells (H). P1 primers (top), P2 primers (middle) and β‐actin primers (bottom). KD, knockdown; NC, negative control; OV, over‐expression.

Now ANXA2 knockdown and ectopic over‐expression were performed in A172 (the identical expression pattern of B7‐H3 in U251MG), LN229, U87MG and U373MG (Figure 2A–D). We surprisingly observed that ANXA2 knockdown caused the absence of B7‐H3 whatever the isoforms were (Figure 2A–C), and supplement of ANXA2 could salvage the expression of B7H3 in U373MG (Figure 2D), indicating that ANXA2 was essential for B7‐H3 isoform maintenance. By contrary, over‐expression of ANXA2 failed to determine the choice of 4Ig or 2Ig in glioma cells.

Moreover, from the transcription profile of B7‐H3, it could be seen that the changes of RNA were largely consistent with the protein in these four glioma cell lines (Figure 2E–H), implying that ANXA2 might affect the process of RNA splicing rather than translation. The only difference was that transcript of 3Ig was not affected by ANXA2.

### 3.3 NSUN2 is required for generation of 2Ig isoform of B7‐H3 in glioma cells

To explore the role in isoform splicing of B7‐H3, we traced back to mass spectrum (Figure 1E). We observed that NSUN2 bound with transcripts of B7‐H3 in LN229, U87MG and U373MG, but not in A172 and U251MG. NSUN2 appeared in the glioma cells expressing 2Ig, but disappeared in cells expressing 4Ig, which aroused our concern. Coincidentally, the presence of the weakened expression of NSUN2 in glioma cells (Figure 1C) and tissues (Figure 1D) with robust abundance of 2Ig, suggesting that the abundance of NSUN2 might be related to the production of 2Ig.

**FIGURE 3:**
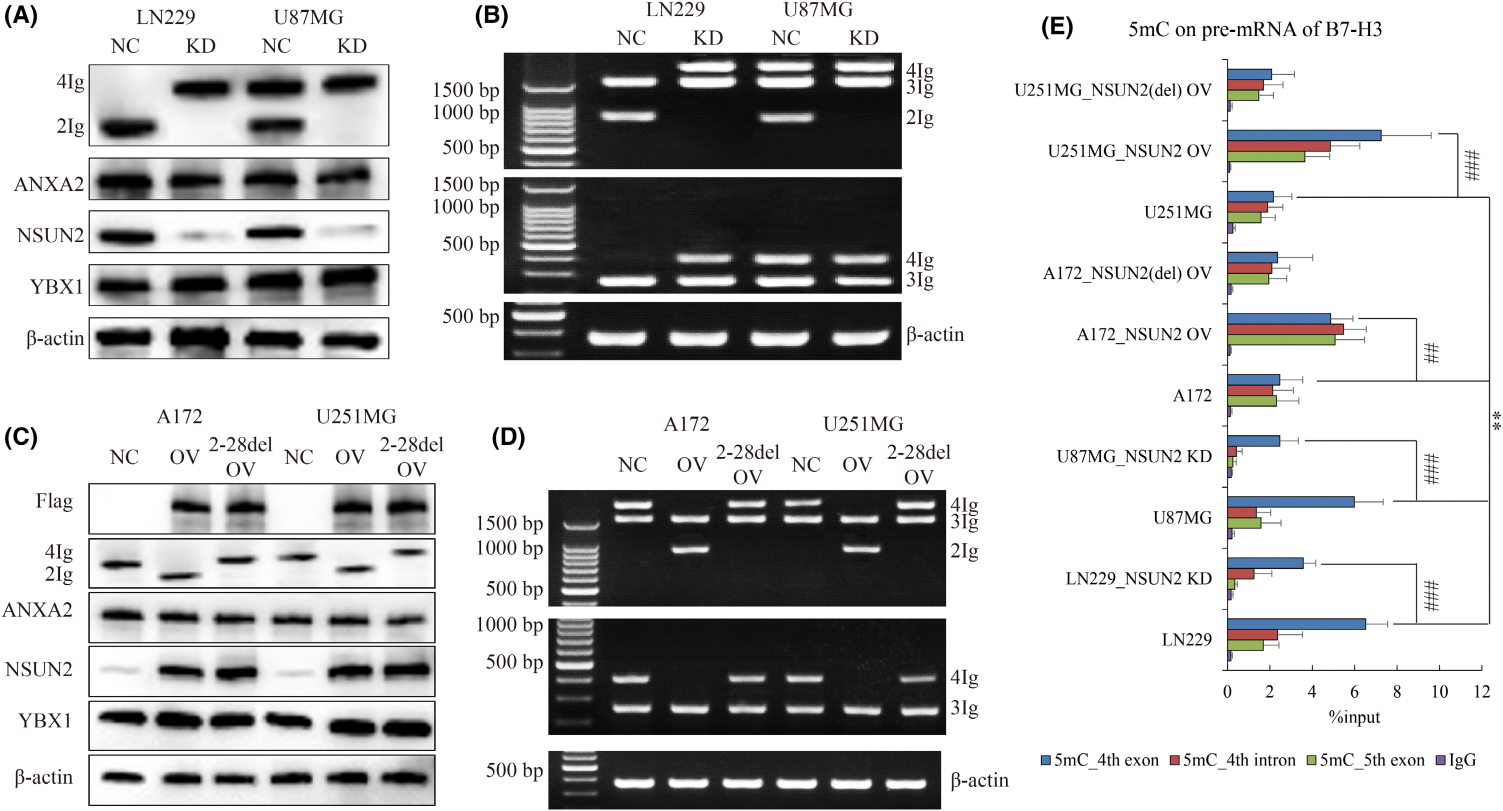
The effect of NSUN2 on the expression of different B7‐H3 isoforms. (A) WB assays show the expression of 4Ig and 2Ig affected by knockdown of NSUN2 in LN229 and U87MG cells. ANXA2 and YBX1 are not affected by NSUN2 knockdown. (B) RT‐PCR show the PCR products of B7‐H3 isoform affected by knockdown of NSUN2 in LN229 and U87MG cells. P1 primers (top), P2 primers (middle) and β‐actin primers (bottom). (C) WB assays show the expression of 4Ig and 2Ig affected by over‐expression of wild type or mutant (deletion of 2‐28 amino acids) NSUN2 in A172 and U251MG cells. ANXA2 and YBX1 are not affected by NSUN2 over‐expression. (D) RT‐PCR show the PCR products of B7‐H3 isoform affected by over‐expression of wild type or mutant NSUN2 in A172 and U251MG cells. P1 primers (top), P2 primers (middle) and β‐actin primers (bottom). (E) Enrichment of 5mC on B7‐H3 4th exon in four glioma cells by meRIP‐qPCR. The statistical significances compared between NSUN2 knockdown or over‐expression and control in each cell line via one‐way ANOVA are indicated by well number. The statistical significances compared between wild‐type cells of LN229, U87MG and A172, U251MG via one‐way ANOVA are indicated by asterisk. Triple and double repetitions of symbols mean *p* value less than 0.001 and 0.01. 2‐28del OV, over‐expression of mutant NSUN2; KD, knockdown; NC, negative control; OV, over‐expression of wild‐type NSUN2.

Logically, NSUN2 knockdown was carried on in LN229 and U87MG cells. We noticed that 2Ig was obviously lost by NSUN2 knockdown in LN229 and U87MG cells (Figure 3A). More surprisingly, 4Ig was generated in LN229 by NSUN2 knockdown (Figure 3A). Consistently, RT‐PCR also verified the altered pattern of B7‐H3 isoforms in these two cell lines (Figure 3B). In turn, ectopic over‐expression of NSUN2 was also carried on in A172 and U251MG cells. Supplementary of NSUN2 could substantially rescue 2Ig in A172 and U251MG cells (Figure 3C). Identically, RT‐PCR showed that wild type NSUN2 could elevate the product of 2Ig isoform in A172 and U251MG cells (Figure 3D). Notably, NSUN2 knockdown or over‐expression neither affected the generation of 3Ig or the expression of ANXA2.

Because NSUN2 primarily acts as a 5‐methyltransferase for RNA, whether NSUN2‐mediated 2Ig of B7‐H3 selection in glioma was modulated via RNA methylation needs to be addressed. Therefore, meRIP‐qPCR was performed to investigate the pattern of methylation of B7‐H3 transcripts. We noticed that the difference between 4Ig and 2Ig transcripts started from 4th exon. Unlike 4Ig, 2Ig transcript lost 19 nucleotides at the end of 4th exon then direct linked with 7th exon to skip 5th and 6th ones. We found that pre‐mRNA of B7‐H3 had higher methylation around the 4th exon in the original cells of LN229 and U87 cells than A172, U251MG (Figure 3E). Furthermore, the abundance of RNA methylation on B7‐H3 could be compromised by NSUN2 knockdown or elevated by NSUN2 over‐expression (Figure 3E).

Additionally, the key fraction (2–28 peptide) of NSUN2 contributed to the activity of NSUN,^23^ over‐expression of NSUN2 with 2–28 truncation failed to control the isoform of B7‐H3 (Figure 3C) or affect RNA methylation (Figure 3E) compared to wild type NSUN2 in A172 and U251MG cells.

### 3.4 YBX1 enhances the production of 2Ig transcript from the methylated B7‐H3 pre‐mRNA

Although the introduction of NSUN2 could well explain the preferential generation of 2Ig B7‐H3 in LN229, U87MG, A172 and U251MG glioma cells, it was still unexplainable that why the wild type U87MG cells had both 4Ig and 2Ig B7‐H3 isomers compared with LN229, even though they both had robust abundance of NSUN2 (Figure 1C). Since NSUN2 exerts as a 5mC “writer”, it was speculated that the proteins related with 5mC “reader” or “eraser” were supposed to influence the biological behaviour of NSUN2 for B7‐H3 isoform selection. Coincidentally, YBX1^24^, ^25^ as a 5mC “reader” displayed a deficient interaction with B7‐H3 transcripts in U87MG (Figure 1E), and the expression of YBX1 was weak in U87MG compared to other four glioma cells (Figure 1C), but was strong in glioma specimens with abundant expression of 2Ig (Figure 1D).

**FIGURE 4:**
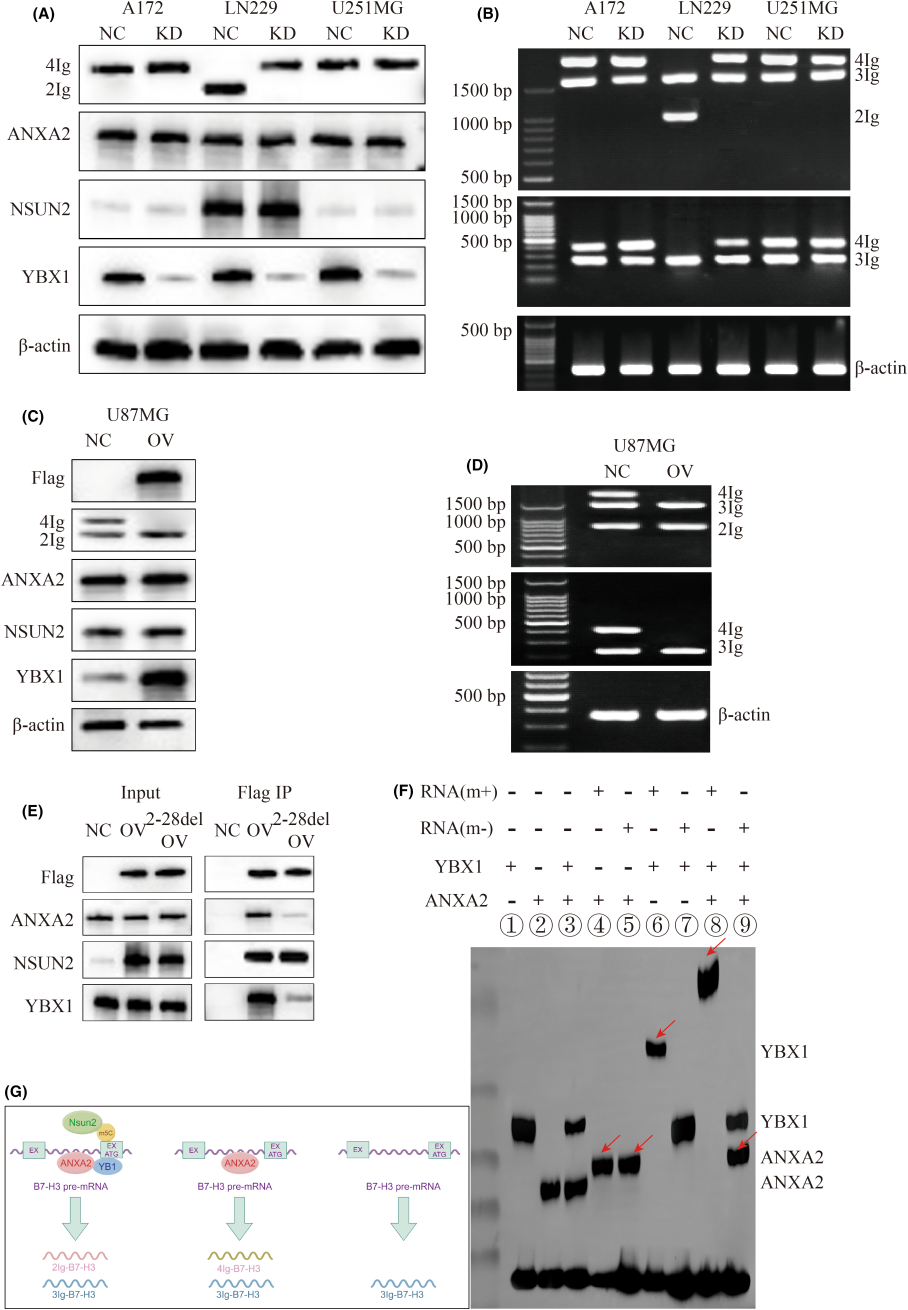
The effect of YBX1 on the expression of different B7‐H3 isoforms. (A) WB assays show the expression of 4Ig and 2Ig affected by knockdown of YBX1 in A172, LN229 and U251MG cells. ANXA2 and NSUN2 are not affected by YBX1 knockdown. (B) RT‐PCR show the PCR products of B7‐H3 isoform affected by knockdown of YBX1 in A172, LN229 and U251MG cells. P1 primers (top), P2 primers (middle) and β‐actin primers (bottom). (C) WB assays show the expression of 4Ig and 2Ig affected by over‐expression of YBX1 in U87MG cell. ANXA2 and NSUN2 are not affected by over‐expression of YBX1. (D) RT‐PCR show the PCR products of B7‐H3 isoform affected by over‐expression of YBX1 in U87MG cell. P1 primers (top), P2 primers (middle) and β‐actin primers (bottom). (E) WB assays show the substantial interaction with ANXA2 and YBX1 by Flag (ectopic NSUN2) IP in A172 cell. Mutant NSUN2 captures much less ANXA2 and YBX1 compared to wild‐type one. (F) RNA‐EMSA shows the interaction among recombinant human YBX1, ANXA2 and the synthetic partial B7‐H3 RNA with or without 5mC in vitro. ANXA2 appears to have slower mobility due to attaching with RNA in Lane 4, 5 and 9. YBX1 appears to have slower mobility due to attaching with methylated RNA in Lane 6. YBX1 and ANXA2 appear to form one complex and show slowest mobility. All these changes are highlighted by red arrows. (G) Schematic representation shows the regulatory relationship between ANXA2/NSUN2/YBX1 on generation of B7‐H3 isoforms in glioma. In accordance with copyright requirements, we hereby declare that this image is drawn using “FIGDRAW” software. m+, methylated RNA; m−: unmethylated RNA.

Similar to the above approach, we continued to perform YBX1 knockdown or supplementation on glioma cells. Our data suggested that YBX1 knockdown also caused the shift from 2Ig to 4Ig in LN229 cells, while could not change 4Ig (Figure 4A,B), and YBX1 over‐expression could lead to 4Ig loss in U87MG cells (Figure 4C,D). We noticed that YBX1 was supposed to be crucial for 2Ig generation, but dependent on enough abundance of NSUN2. For example, in wild type A172 and U251MG, because the expression of NSUN2 is very low, whether YBX1 knockdown does not affect the shift between 4Ig and 2Ig (Figure 4A).

#### 3.4.1 Coordination of ANXA2, NSUN2 and YBX1

Finally, the interplay among ANXA2, NSUN2 and YBX1 was performed by Flag (NSUN2) immunoprecipitation in A172 cells. We observed that endogenous ANXA2 and YBX1 could be recruited together only by the active NSUN2 replenishment (Figure 4E).

To verify once again the role of NSUN2 function in this regulatory complex, NSUN2 knockdown ex vivo was replaced by B7‐H3 transcript with or without 5mC modification in vitro assay. The RNA molecule of the 4nd exon and 4nd intron of B7‐H3 pre‐mRNA was synthesized, and put together with the recombinant human ANXA2 and YBX1 for RNA‐EMSA (Figure 4F).

In lane 4, ANXA2 could not bind with RNA due to the absence of YBX1. While in lane 6, the slower mobility of RNA suggested that YBX1 was able to bind with methylated RNA even without ANXA2. In lane 7, YBX1 failed to interact with unmethylated RNA. In lane 8, the slowest mobility compared to lane 6 indicated that ANXA2 and YBX1 both interacted with methylated RNA. These results in vitro again indicated that 5mC modification on B7‐H3 transcripts was necessary for YBX1 recognition, as well as YBX1 binding to B7‐H3 was essential for ANXA2 recruitment.

Taken together, our study provided an explainable theory that ANXA2/NSUN2/YBX1 axis modulated the generation of B7‐H3 isoform in glioma cells (Figure 4G).

## 4 DISCUSSION

The phenotypes of 4Ig and 2Ig in glioma are unclear, and the regulatory molecular mechanism is still unknown, which is unfavourable to the CAR‐T target design of CD276 for glioma immunotherapy. Our observations in cell lines and in vivo both suggested a distinct and complicated phenotype of B7‐H3 (Figure 1B–D). Here, our findings suggest that when studying B7‐H3, the materials used, such as glioma cell lines or case specimens, and even generalized to other tumours, need to pay special attention to the expression of different isoforms of B7‐H3, because their different structures are likely to lead to the completely different conclusions. For example, it has been suggested that 4Ig rather than 2Ig can form dimers,^26^ and increase the activation of AKT, JAK/STAT, HIF1α, NF‐κβ for cell proliferation.^27^ The CAR‐T targets currently in use are also designed for 4Ig.^28^, ^29^ Although 4Ig is dominant in glioma tissues of the few glioma specimens we collected, 2Ig is specially expressed in glioma patients with malignant grade, hinting that designing CAR‐T targets for 2Ig may be a new strategy for high‐malignant glioma.

The most important method in this paper is to capture the endogenous pre‐mRNA of B7‐H3 by probes to examine the RBPs in different cell lines. By comparing among five glioma cells, we have finally found three proteins ANXA2/NSUN2/YBX1 that can explain the phenotypes in different cells. ANXA2 is a protein with clear evidence to play an important role in alternative splicing for numerous malignancies and immune host response.^30^, ^31^, ^32^ NSUN2 and YBX1 are both important regulatory proteins in the RNA methylation system. The presence of the facilitation of 2Ig expression by YBX1 and the following positive correlation with high‐malignant glioma in this study is consistent with previously studies that high expression of YBX1 appears to the carcinogenic effect.^33^, ^34^ Similarly, the suppression of tumour metastasis by NSUN2 knockdown^35^ also suggests an oncogenic effect of NSUN2 in glioma. The axis of NSUN2/YBX1 can promote the generation of 2Ig of B7‐H3 in glioma, and we speculate that the abundant 2Ig takes up too much of the cell surface, causing the T cells to fail to recognize enough 4Ig, allowing the glioma to become immune escape capability. We believe that the illustration of ANXA2/NSUN2/YBX1 regulatory axis is a prerequisite to address the complicated pattern of B7‐H3 in glioma, which again highlights the problem of immunotherapy not being universally effective in the cancer population.

Of interest in our study of the B7H3 phenotype is U373MG cell, it is negatively expressed B7‐H3 (Figure 1B,C); whereas, the similar phenotype was not observed in specimens in vivo due to the limited sample size. Nevertheless, the possibility of encountering clinically negative CD276 glioma patients is worthy of our serious thinking about new solutions beyond CAR‐T against CD276. Another confusing object is 3Ig, which has never been reported in previous literature. This transcript is stably expressed in all glioma cells but never translated into protein nor affected by ANXA2. In our study, we cannot address the significance and potential functions of 3Ig for B7‐H3 in glioma.

The shortcomings of this study include two other points: (1) the therapeutic effect of 4Ig and 2Ig‐targeted CAR‐T at least in vitro is not compared; (2) the biochemical principle of alternative splicing of B7‐H3 is not clarified. These two issues will be addressed in our future research plan.

## AUTHOR CONTRIBUTIONS

**Yifen Shen:** Investigation (equal); methodology (lead); validation (lead); visualization (lead). **Chunfang Ma:** Software (equal). **Xiangxiang Li:** Investigation (equal); validation (equal); visualization (equal). **Xiaosong Li:** Investigation (equal); validation (equal); visualization (equal). **Yuxiang Wu:** Investigation (equal); validation (equal); visualization (equal). **Tao Yang:** Investigation (equal); validation (equal); visualization (equal). **Yanping Hu:** Methodology (equal); resources (equal). **Chao Liu:** Funding acquisition (equal); resources (equal). **Hao Shen:** Funding acquisition (equal); resources (equal). **Pin Guo:** Funding acquisition (lead); resources (lead). **Yihang Shen:** Conceptualization (lead); funding acquisition (equal); investigation (equal); project administration (lead); validation (lead); visualization (lead); writing – original draft (lead); writing – review and editing (lead).

## FUNDING INFORMATION

This project was supported by Jiangsu Province High‐level Innovation and Entrepreneurship Talent Plan (JSSCBS20222022), Science and Technology Planning Fund of Affiliated Hospital of Xuzhou Medical University (XYFY202219, XYFY202306, XYFY202307), Youth Program of Developing Public Health through Science and Education of Suzhou (KJXW2023081), Gusu Health Talent Training Project (GSWS2019087), the People's Livelihood Science and Technology of Suzhou (SYSD2020043), Suzhou Science and Technology Planning Project (SKYD2022023), Program of Developing Public Health through Science and Education of Wujiang District, Suzhou (wwk202306), General Program of Natural Science Foundation of Shandong Province (ZR2020MH261), and Project of Suzhou Ninth People's Hospital (YK202226, YK202425).

## CONFLICT OF INTEREST STATEMENT

The authors declare no conflict of interests.

## CONSENT FOR PUBLICATION

Informed consent forms were signed by all patients and their families.

## Data Availability

Data will be made available on request.
